# The Fitness of Beta-Lactamase Mutants Depends Nonlinearly on Resistance Level at Sublethal Antibiotic Concentrations

**DOI:** 10.1128/mbio.00098-23

**Published:** 2023-04-27

**Authors:** Andrew D. Farr, Diego Pesce, Suman G. Das, Mark P. Zwart, J. Arjan G. M. de Visser

**Affiliations:** a Laboratory of Genetics, Wageningen University & Research, Wageningen, The Netherlands; b Department of Microbial Population Biology, Max Planck Institute for Evolutionary Biology, Plön, Germany; c Institute for Biological Physics, University of Cologne, Cologne, Germany; d Department of Microbial Ecology, Netherlands Institute of Ecology (NIOO-KNAW), Wageningen, The Netherlands; University of Pittsburgh School of Medicine

**Keywords:** fitness landscape, subinhibitory antibiotics, sub-MIC, TEM-1 β-lactamase, cefotaxime, amplicon sequencing, filamentation

## Abstract

Adaptive evolutionary processes are constrained by the availability of mutations which cause a fitness benefit and together make up the fitness landscape, which maps genotype space onto fitness under specified conditions. Experimentally derived fitness landscapes have demonstrated a predictability to evolution by identifying limited “mutational routes” that evolution by natural selection may take between low and high-fitness genotypes. However, such studies often utilize indirect measures to determine fitness. We estimated the competitive fitness of mutants relative to all single-mutation neighbors to describe the fitness landscape of three mutations in a β-lactamase enzyme. Fitness assays were performed at sublethal concentrations of the antibiotic cefotaxime in a structured and unstructured environment. In the unstructured environment, the antibiotic selected for higher-resistance types—but with an equivalent fitness for a subset of mutants, despite substantial variation in resistance—resulting in a stratified fitness landscape. In contrast, in a structured environment with a low antibiotic concentration, antibiotic-susceptible genotypes had a relative fitness advantage, which was associated with antibiotic-induced filamentation. These results cast doubt that highly resistant genotypes have a unique selective advantage in environments with subinhibitory concentrations of antibiotics and demonstrate that direct fitness measures are required for meaningful predictions of the accessibility of evolutionary routes.

## INTRODUCTION

The mapping of genotypic space with fitness is a primary concern of evolutionary biology. This interest arose because this mapping bridges genetics and evolution, being shaped by the interactions between mutations and governing which mutational trajectories evolution can follow. Since 1932 (1), the “fitness landscape” metaphor has been used to visualize the complicated mapping of genotype to fitness. More recently, molecular biology has made possible the construction of mutational networks in which sets of mutations—in either one (2–9) or multiple (10–14) genes—are combined and expressed in an experimental strain. The interactions among mutations generate the topography of these empirical fitness landscapes and render particular trajectories across the landscape more likely than others. In a foundational study, Weinreich and colleagues demonstrated that of the 120 possible trajectories linking an ancestral β-lactamase antibiotic resistance allele with a highly adapted genotype with five mutations, only 18 trajectories are selectively accessible (3). The level of constraint in such landscapes suggests a degree of predictability of evolutionary processes, although the relation between epistatic constraints and evolutionary predictability is complicated by the presence of multiple adaptive peaks (15, 16).

For empirical fitness landscapes to inform evolutionary biology and enable meaningful predictions regarding evolutionary processes, they require informative relative fitness measures of each genotype in the landscape. However, the actual measures of fitness used to form such fitness landscapes generally do not represent the outcome of direct competition between genotypes. In real life, *de novo* variants often directly compete with their progenitors, making it essential to use fitness measures that capture their competitive ability to predict their fates. Despite their name, empirical fitness landscapes are often quantified by the functionality of a focal enzyme. The mutational networks in dihydrofolate reductase (2), methyl-parathion hydrolase (17), and β-lactamase (3, 5, 8, 18) have used the activity of the enzymes and their effects on organismal growth or survival as proxies for fitness. Several empirical fitness landscapes do provide measures of the relative fitness of genotypes. However, with some notable exceptions (19), these fitness measures are made relative to a common competitor—not immediate evolutionary predecessors—and may be complicated by nontransitive interactions among the genotypes, causing deformations of the landscape due to ecoevolutionary changes of the environment (20). Such environmental alterations may occur if genotypes alter the extracellular concentration of a common resource or inhibitory substance available to cocultivated genotypes (21–24). Although technically challenging, fitness landscapes would ideally present fitness measures of each genotype relative to mutational neighbors from which they arise.

**FIG 1:**
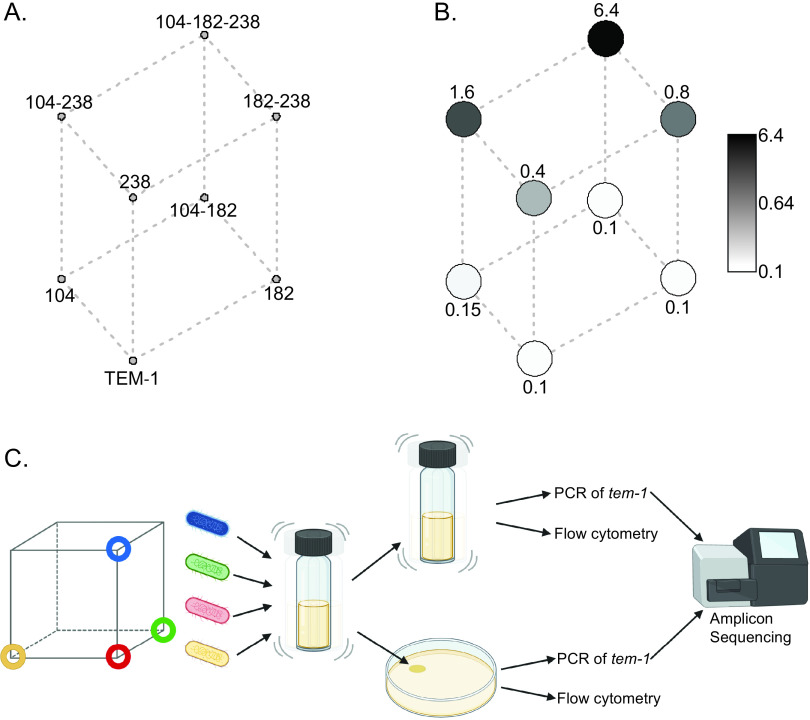
Levels of resistance across the 8-genotype landscape of TEM-1 β-lactamase for cefotaxime resistance and the method used in measuring fitness. (A) Cube diagram of the 8-node mutations (numbers refer to amino acid positions of substitutions) with (B) grayscale circles overlaid representing median MIC measures in µg mL-1 of CTX (see table 1). (C) Summary of the experimental design to measuring relative fitness of genotypes in the landscape. A focal genotype and the three mutational neighbors with a Hamming distance of 1 (each represented by a different color) were each cultured and mixed in equal ratios (*T*_0_ sample). The mixture of genotypes was then either inoculated into liquid M9 medium or spotted onto M9 medium solidified with agar either without or with 0.02 or 0.04 μg mL^−1^ of CTX. Competition mixtures were sampled at 0, 24, and 48 h, whereupon cell concentrations were measured by flow cytometry, or samples were stored for PCR of *Tem-1* followed by amplicon sequencing to determine the ratio of mutations present.

We constructed an empirical fitness landscape measured by the competitive fitness of each genotype in the landscape relative to its immediate mutational neighborhood. This landscape consists of combinations of three mutations which underpin the evolution of the β-lactamase TEM-52, a clinically relevant member of the extended-spectrum β-lactamases (ESBLs). Following the clinical introduction of cephalosporin antibiotics, ESBLs were selected with the capacity to bind and hydrolyze cephalosporins such as cefotaxime (CTX), resulting in genotypes such as TEM-52. TEM-52 evolved from the ancestral TEM-1 β-lactamase, which effectively degrades β-lactams such as ampicillin but has low activity against cefotaxime (25). The evolution of TEM-52 from TEM-1 involved three nonsynonymous mutations, causing amino acid substitutions E104K, M182T, and G238S, which in various combinations form an eight-genotype mutational landscape (Fig. 1A).

To assess the adaptive benefit of the mutations across the landscape, this set of eight genotypes was competed with its three neighboring genotypes with a Hamming distance of one. Competitions were performed in a structured (on the surface of agar-solidified medium) and an unstructured (in liquid medium) environment, to investigate the role of spatial structuring in the fitness of competitors. Spatially structured environments—such as colonies grown on agar—were previously shown by Frost and colleagues (26) to provide a selective benefit to nonresistant strains when cocultured with resistant strains. This benefit was due to the induction in sensitive strains of filamentous cellular morphologies, a physiological response of sensitive bacteria exposed to near-inhibitory concentrations of β-lactam antibiotics (27). These elongated cells helped the nonresistant strains to invade the unoccupied space of the colony edge, where they presumably had better access to resources, ultimately resulting in a selective benefit over nonelongated resistant strains (26). The reproducibility of this phenomenon was assessed in our experimental system, as was how spatial structuring alters the topography of the fitness landscape. Our fitness assays were performed with subinhibitory concentrations of CTX, defined here as the range of antibiotic concentrations which permit monocultures of a genotype to grow. The choice of these low concentrations was driven by two considerations: (i) the practical need to measure fitness requires the presence of competitors after a growth period, and high CTX concentrations may completely eliminate low-resistance competitors, and (ii) the growing interest in subinhibitory concentrations of antibiotic as representative of concentrations in environmental settings such as wastewater and sediment (28–30) and the potential for such subinhibitory concentrations to select for resistant genotypes (14, 31–34). When competed in these subinhibitory concentrations, our set of genotypes displayed fitness landscapes that were highly conditional on both antibiotic concentration and spatial structuring of the environment.

## RESULTS

### Experimental model: the TEM-1 to TEM-52 mutational landscape.

The genotypes studied were made by insertion of TEM-1 alleles, reconstructed with all combinations of the three mutations that comprise TEM-52, into the chromosomal *galK* locus of Escherichia coli MG1655 expressing blue fluorescent protein (BFP) (Table 1; see also Materials and Methods). To compare the enzymatic effects of the resulting mutant enzymes, MIC assays were performed with each biological replicate used in the fitness assays (Table 1 and Fig. 1; see also Materials and Methods). As expected, the magnitude of these resistance measures was consistently lower than previously measured for plasmid-borne TEM alleles (3), likely due to gene dosage arising from the single copy of this gene in the chromosome. In keeping with other studies on the enzymatic consequences of these mutations (3, 5), particular combinations of mutations caused a much higher level of resistance than others (Fig. 1). Of the single mutations, the G238S amino acid change causes a large increase in resistance, and similarly, the E104K-G238S mutant is the most resistant of the double mutants. The E104K-M182T-G238S triple mutant confers the highest level of resistance of this landscape of mutants.

**TABLE 1:** Strains used in this study and associated MIC values^*a*^

Strain	Genotype	Median MIC (μg mL^−1^)	Reference
DA28200	E. coli MG1655 *galK*::SYFP2-FRT	0.1	32
DA28202	E. coli MG1655 *galK*::mTagBFP2-FRT (parent of strains below)	0.04	32
TEM-1	*galk*::*TEM-1*	0.1	This study
M182T mutant	*galk*::*TEM-1* M182T	0.1	This study
E104K mutant	*galk*::*TEM-1* E104K	0.15	This study
G238S mutant	*galk*::*TEM-1* G238S	0.4	This study
M182T-E104K mutant	*galk*::*TEM-1* M182T E104K	0.1	This study
M182T-G238S mutant	*galk*::*TEM-1* M182T G238S	0.8	This study
E104K-G238S mutant	*galk*::*TEM-1* E104K G238S	1.6	This study
E104K-M182T-G238S mutant	*galk*::*TEM-1* E104K M182T G238S	6.4	This study

a DA28202 was the parent strain for all subsequent TEM-1 mutations used in this study. DA28202 expresses BFP, allowing cellular counts to be made by flow cytometry. Median MIC assays of the TEM-1 mutants were performed on all four biological replicates used in the landscape fitness assays. The median MICs of DA28200 and DA28202 were measured by visual observation, and median MICs of the TEM-1 genotypes were determined at a separate occasion using OD_600_ measures of growth (see Materials and Methods).

### Paired competitions describe inverted and stratified resistance-fitness relationships depending on the environment.

**FIG 2:**
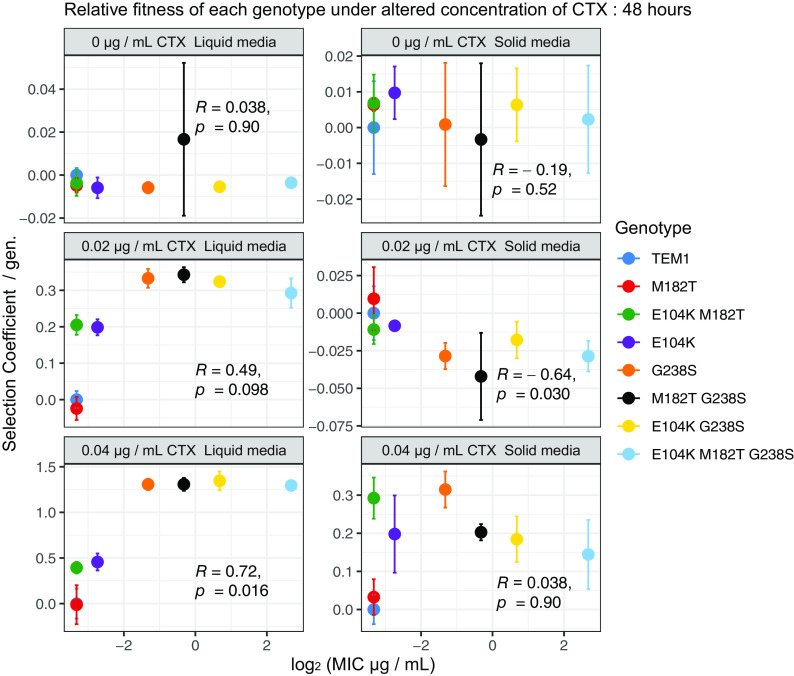
Selection coefficient of β-lactamase mutants (relative to strain MG1655-YFP) relative to the log_2_ MIC of each genotype. Relative to a common competitor (not expressing TEM-1), the selection coefficients of TEM-1 mutants were significantly correlated with resistance under some competitive conditions. In liquid medium, more resistant types exhibited higher selection coefficient values, which was significant at 0.04 μg mL^−1^ of CTX. Competed on solid medium, higher-resistance types had significantly lower selection coefficient values at 0.02 μg mL^−1^ of CTX. Data points represent the means of three biological replicates, error bars represent standard deviations, and statistical test values presented are Kendall rank correlation coefficient rho and *P* values for the correlation of selection coefficient and MIC values of each genotype.

The effect these mutations have on relative fitness was initially assessed by pairwise competition. Each of the eight genotypes of the landscape was competed with a common reference strain labeled with chromosomally encoded yellow fluorescent protein (YFP) which did not express TEM-1. Fitness assays were performed in structured (agar media) and unstructured (liquid media) environments containing 0, 0.005, 0.01, 0.02, 0.04, and 0.08 μg mL^−1^ of CTX, and cell numbers were measured by flow cytometry (see Materials and Methods). Pairwise fitness assays of these strains in the absence of CTX revealed no significant relationship between the alleles’ ranked resistance and relative fitness; hence, the alleles showed no trade-off between resistance and competitive fitness (Fig. 2 and Fig. S1 and S2). Not surprisingly, expression of higher-resistance alleles was beneficial in the unstructured environments supplemented with 0.005 μg mL^−1^ to 0.08 μg mL^−1^ of CTX. This observation was pronounced for all genotypes featuring the G238S mutation. At both 0.02 μg mL^−1^ and 0.04 μg mL^−1^, all genotypes which include the G238S mutation had similar mean selection coefficients, despite an approximate 16-fold difference in the median MICs of the G238S and E104K-M182T-G238S mutants (Table 1).

10.1128/mbio.00098-23.1 FIG S1 Selection coefficients of TEM-1 mutants (competed with MG1655-YFP in liquid media) relative to the log_2_ MIC of each genotype. Data points represent the means of three biological replicates, error bars represent standard deviations, and statistical test values presented are Kendall rank correlation coefficient rho and *P* values for the corelate of selection coefficient and MIC values for the genotypes in each plot. Download FIG S1, PDF file, 0.3 MB. Copyright © 2023 Farr et al. 2023 Farr et al. https://creativecommons.org/licenses/by/4.0/ This content is distributed under the terms of the Creative Commons Attribution 4.0 International license.

10.1128/mbio.00098-23.2 FIG S2 Selection coefficients of TEM-1 mutants (competed with MG1655-YFP on solid media) relative to the log_2_ MIC of each genotype. Competitions with 0.08 μg mL^−1^ of CTX resulted in null cell counts of either competitor for at least one replicate, and resulting means were not plotted. Number of replicates, error bars, and presented statistical test values are identical to those in Fig. S1. Download FIG S2, PDF file, 0.3 MB. Copyright © 2023 Farr et al. 2023 Farr et al. https://creativecommons.org/licenses/by/4.0/ This content is distributed under the terms of the Creative Commons Attribution 4.0 International license.

In structured environments, the relationship between resistance and relative fitness was more complex. Fitness differences among genotypes was smaller than in liquid cultures (Fig. 2). After 24 and 48 h of competition in 0.005, 0.01, and 0.02 μg mL^−1^ of CTX, the resistance of genotypes was significantly negatively correlated with fitness (Fig. S2). Assays performed at 0.04 μg mL^−1^ no longer showed a negative relationship between resistance and fitness, but no significant positive relationship was observed after either 24 or 48 h of competition (Fig. 2 and Fig. S2). The highest concentration, 0.08 μg mL^−1^, of CTX resulted in low or no recovery of low resistance competitors, and multiple-genotype competitions (see below) were conducted at lower concentrations of CTX, 0.02 and 0.04 μg mL^−1^.

### Relative fitness of each genotype relative to its three single-mutation neighbors.

Direct measures of fitness between neighboring genotypes of the 8-mutant fitness landscape were made. Each of the eight genotypes was competed with the three neighboring genotypes, resulting in eight competitions involving four competitors (Fig. 1C). Fitness assays were performed at three concentrations of CTX: at the concentration where sensitive genotypes had a growth disadvantage in pairwise comparisons but were still recoverable (0.04 μg mL^−1^), at half this concentration (0.02 μg mL^−1^), and in environments without CTX. Amplicon sequencing was used to determine the relative fitness of four competitors simultaneously. Deep-sequencing data were analyzed with an approach insensitive to the effects of recombination between TEM alleles, caused by low-rate template switching during PCR amplification (see Materials and Methods). In order to derive selection coefficients, the total inoculum size and final yield were derived using flow cytometry (Fig. 1C).

**FIG 3:**
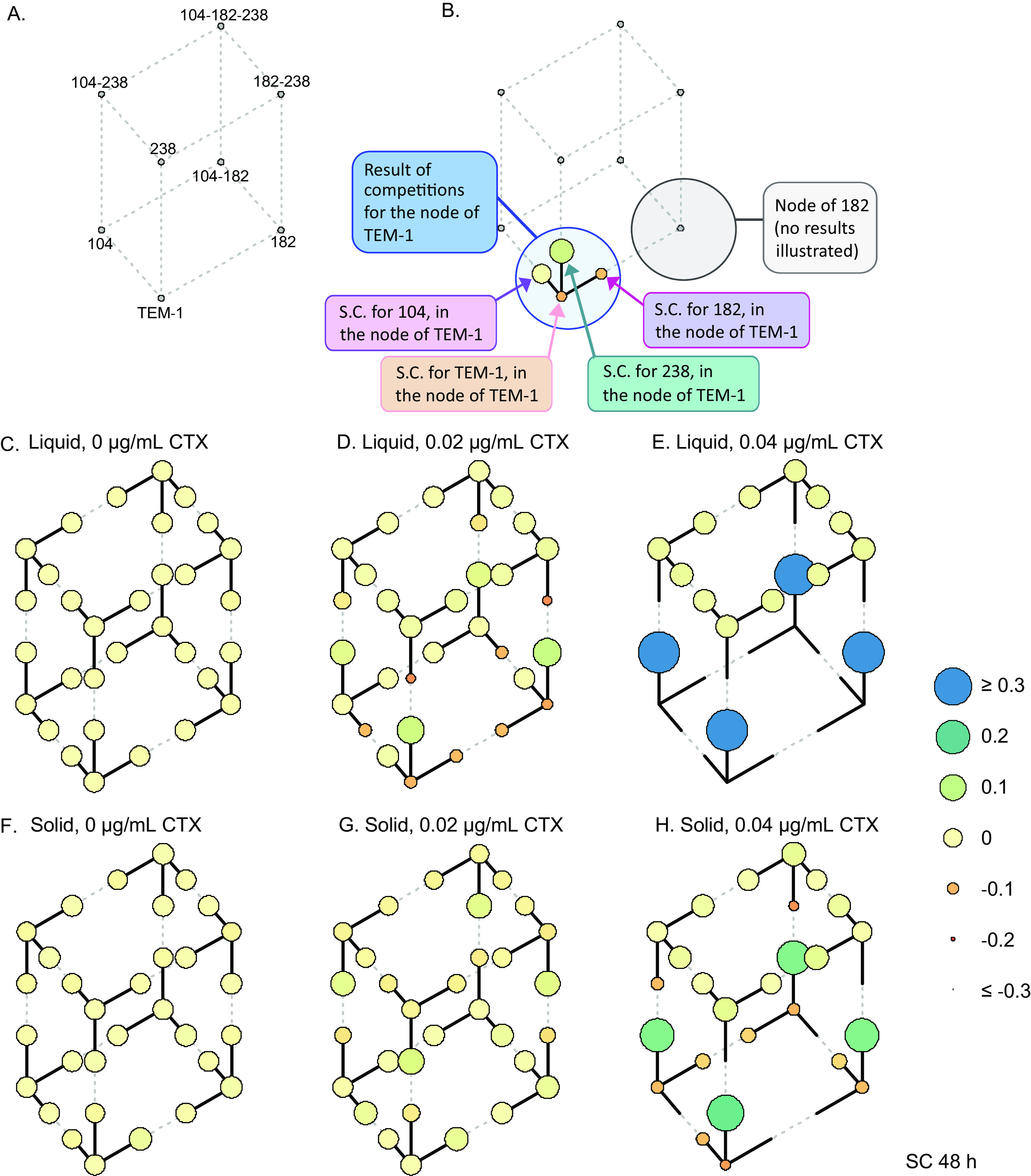
Selection coefficients (SC) of each genotype relative to three genetically related competitors. (A) The network of eight genotypes in this study. (B) Guide illustrating the results of one competition for the node TEM-1. A total of eight competitions were performed—one competition per node—each involving a focal strain and the three competitors distinct from the “focal” strain by a single mutation. For instance, the node of TEM-1 was comprised of a competition of the focal strain TEM-1, together with E104K, M182T, and G238S. (C) Selection coefficient data of 8 genotypes, each measured relative to three competitors at 48 h. Competitions were performed in either liquid (C to E) or solid media (F to H) with either 0, 0.02, or 0.04 μg mL^−1^ of CTX. SC values are represented by the size and color of the circles (see key) and are capped to −0.3 and +0.3 to allow visualization of fitness differences at lower CTX concentrations. Each circle represents the mean SC value of four independent replicates.

**FIG 4:**
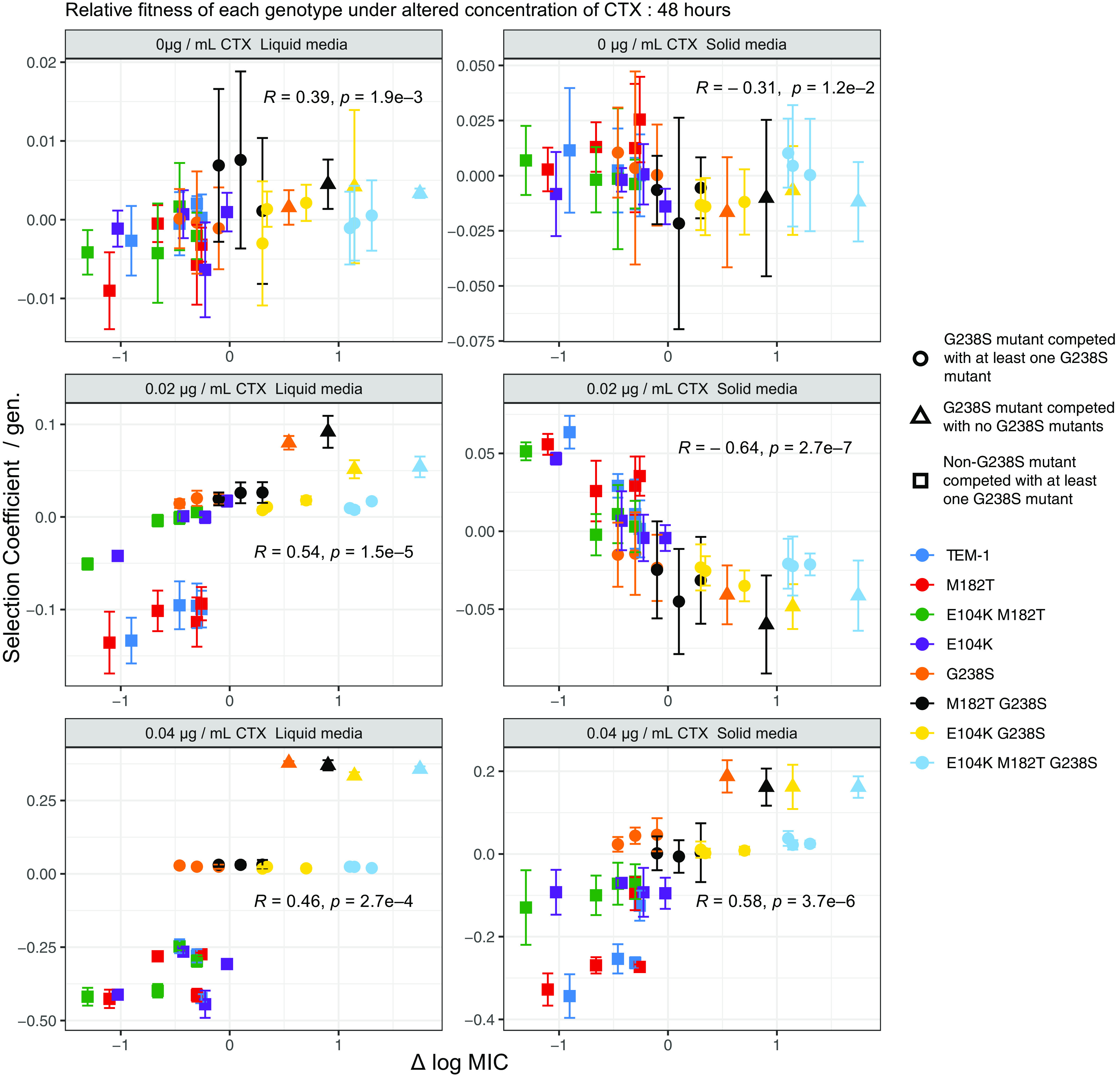
Relationship between selection coefficient and resistance of genotypes relative to their mutational neighbors. Data points represent the mean selection coefficient of each genotype relative to the three other competitors with a Hamming distance of 1 determined over 48 h and the difference between the median log MIC of each genotype and the mean log MIC of the three competitors. A correlation between selection coefficient and resistance was observed at both concentrations of CTX in liquid media, with an increased stratification of selection coefficients at the highest CTX concentration due to the clustering of genotypes with or without mutation G238S, despite differences in resistance. A similar relationship between selection coefficients and resistance was observed at higher concentrations of CTX for competitions on solid medium. However, a significantly negative correlation was observed when genotypes were competed on solid medium at the lower concentration of CTX. See Fig. S4 for similar data at 24 h. Data points represent the means of four biological replicates, error bars represent standard deviations, and statistical test values presented are Kendall rank correlation coefficient rho and *P* values.

The relative fitness of particular genotypes in liquid culture is partially predictable by their resistance measures. At both concentrations of CTX in liquid cultures, expression of the G238S mutation afforded genotypes a clear fitness advantage relative to non-G238S-expressing genotypes (Fig. 3 and Fig. S3). There was generally a positive correlation between resistance and fitness measures of each genotype relative to its three competitors in liquid cultures with 0.02 and 0.04 μg of CTX mL^−1^ (Fig. 4 and Fig. S4). However, this positive correlation of resistance and fitness was not always apparent. At both concentrations of CTX in liquid culture, the four high-resistance genotypes containing mutation G238S had similar fitness measures. To confirm that these four genotypes have similar fitness benefits under the tested conditions, direct selection coefficients were calculated for each of these types relative the other G238S-expressing competitors. Despite substantial differences in resistance between the genotypes, there was no significant relationship between resistance and selection coefficient values of these high-resistance genotypes under any tested condition at either time point (Fig. S5 and S6).

10.1128/mbio.00098-23.3 FIG S3 Mean selection coefficient (SC) measures of each genotype relative to three competitors at 24 h. Figure is identical to Fig. 3 in the main text; however, this supplemental figure presents the earlier time point of 24 h. Each arrow or circle represents the SC of four biological replicates. Measures of error are not included to aid visual clarity. Download FIG S3, PDF file, 0.3 MB. Copyright © 2023 Farr et al. 2023 Farr et al. https://creativecommons.org/licenses/by/4.0/ This content is distributed under the terms of the Creative Commons Attribution 4.0 International license.

10.1128/mbio.00098-23.4 FIG S4 Concentration dependent relationships of resistance and selection coefficient of genotypes competed in 4-genotype competitions over 24 h. The relationship at 24 h is similar to that observed at 48 h, except that at 24 h there is no significant correlation between selection coefficient and resistance in the absence of CTX. Data points represent the means of four biological replicates, error bars represent standard deviations, and statistical test values presented are Kendall rank correlation coefficient rho and *P* values. Download FIG S4, PDF file, 0.2 MB. Copyright © 2023 Farr et al. 2023 Farr et al. https://creativecommons.org/licenses/by/4.0/ This content is distributed under the terms of the Creative Commons Attribution 4.0 International license.

10.1128/mbio.00098-23.5 FIG S5 Relationship of resistance and selection coefficient of genotypes expressing a G238S mutation competed in 4-genotype competitions over 24 h. Kendall rank correlations indicate no significant relationships between selection coefficient and relative resistance under any condition for these genotypes. Data points represent the means of four biological replicates, error bars represent standard deviations, and statistical test values presented are Kendall rank correlation coefficient rho and *P* values. Download FIG S5, PDF file, 0.2 MB. Copyright © 2023 Farr et al. 2023 Farr et al. https://creativecommons.org/licenses/by/4.0/ This content is distributed under the terms of the Creative Commons Attribution 4.0 International license.

10.1128/mbio.00098-23.6 FIG S6 Relationship of resistance and selection coefficient of genotypes expressing a G238S mutation competed in 4-genotype competitions over 48 h. As at 24 h, Kendall rank correlations indicate no significant relationships between selection coefficient and relative resistance under any condition. Number of replicates, error bars, and presented statistical test values are identical to those in Fig. S5. Download FIG S6, PDF file, 0.2 MB. Copyright © 2023 Farr et al. 2023 Farr et al. https://creativecommons.org/licenses/by/4.0/ This content is distributed under the terms of the Creative Commons Attribution 4.0 International license.

Competitions in spatially structured environments resulted in a positive correlation of relative fitness and resistance at the highest CTX concentration (Fig. 4). Similar to the case with the liquid competitions, genotypes expressing the G238S mutation—regardless of additional mutations and level of resistance—had similar fitnesses when directly competed with each other (Fig. S5 and S6). As seen in the pairwise competitions (Fig. 2), there was a significant negative correlation between fitness and resistance of each genotype in structured environments with 0.02 μg mL^−1^ of CTX (Fig. 4). Under these conditions, the mixing of high- and low-resistance types results in selection for low-resistance genotypes.

Additional modeling of the resistance-fitness relationship and simulations of evolutionary trajectories confirm the importance of the large-effect G238S mutation for stratifying the landscape and dictating evolutionary fates (see supplemental text file 1 in the Zenodo repository [35]).

### Cellular filamentation predicts the inverse relationship of resistance and fitness in structured environments.

**FIG 5:**
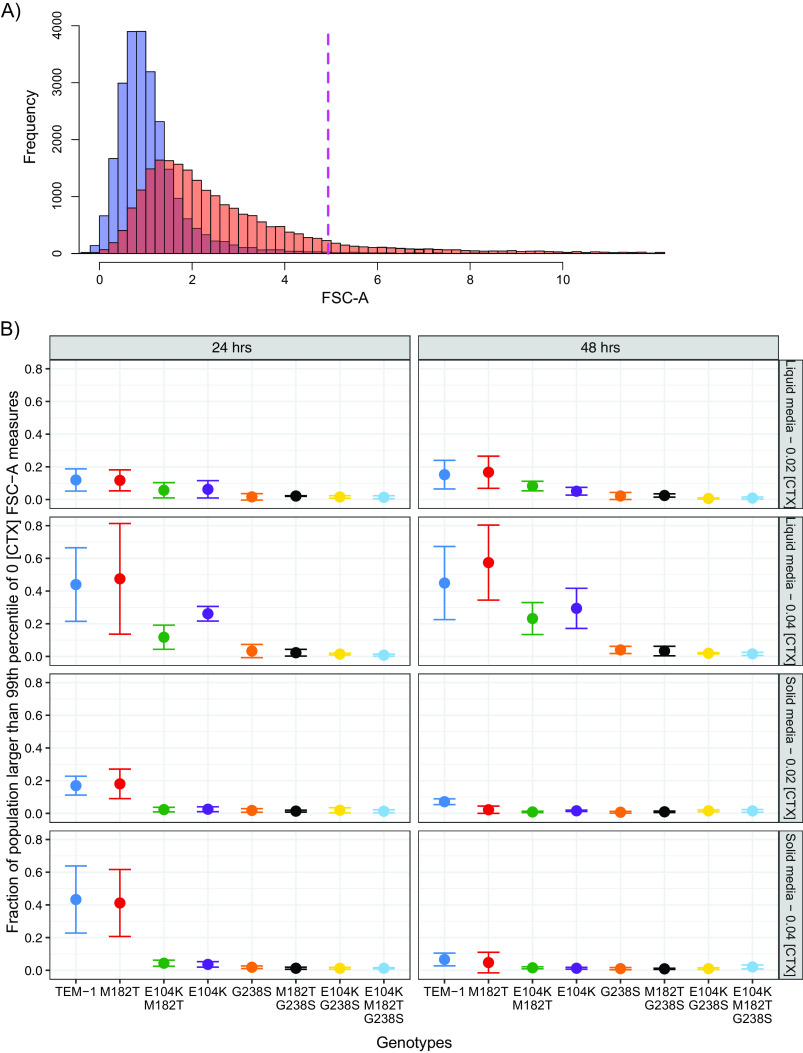
Monocultures of genotypes sensitive to CTX produce larger cells as measured by flow cytometry. (A) Representative histogram of the forward scatter (FSC-A, indicating cellular size) of a single replicate TEM-1 genotype grown in liquid medium supplemented with 0 (blue) or 0.02 (red) μg mL^−1^ of CTX. The dashed red line indicates the upper 99th percentile of cells grown without CTX as counted in the FSC channel. (B) Mean fraction of cells for each genotype grown in monoculture on solid and liquid media supplemented with CTX, exhibiting FSC values larger than the dashed red line in panel A which we refer to as filaments. Measures of cells were taken after 24 and 48 h, with the fraction of filaments declining between 24 and 48 h in populations of less resistant types (TEM-1 and M182T mutant). Data points represent means of three biological replicates; error bars represent standard deviations.

The observation of high relative fitness of low-resistance genotypes in a structured environment at a low CTX concentration prompted explanation. Previous work has identified filamentation of low-resistance types as capable of providing an advantage in direct competitions (26). Flow cytometry measures were used to identify whether our fitness assay conditions were also capable of inducing filamentous cells. To measure the potential of filamentation, each genotype was grown alone in spatially structured and unstructured media with 0, 0.02 and 0.04 μg mL^−1^ of CTX. The degree of filamentation of these monocultures was estimated by observing the distribution of flow cytometry forward scatter area (FSC-A) measures (Fig. 5A), with larger measures suggesting longer cells (36). Three interesting patterns were evident when analyzing these data (Fig. 5B) with a general linear model (Table S1). First, environmental structure affected the fractions of large FSC-A events (*P < *0.001), indicating a higher prevalence of filaments in liquid medium than in solid medium. Second, there was a two-way interaction between ranked resistance and CTX concentration in their effect on filamentation (*P < *10^−4^), indicating that less resistant types tended to filament more at higher CTX concentrations. Third, there was a highly significant three-way interaction between ranked resistance, environmental structure, and time (*P < *10^−9^), capturing the decrease in filamentation on solid medium over time, which occurred only for low-resistance types (Fig. 5B). These results demonstrate that while cellular filamentation is a predictor of low relative fitness in the presence of antibiotics in liquid coculture, in the context of mixed genotypes grown as colonies at subinhibitory concentrations of CTX, filamentation provides a mechanism by which less resistant genotypes hold a fitness advantage over higher-resistance genotypes.

10.1128/mbio.00098-23.7 TABLE S1 General linear model (GLM) for flow cytometry data. We analyzed the log_10_-transformed fraction of FSC-A events which were higher than the 99th percentile for control populations with no antibiotics with a GLM. Variables included in the model were the ranked resistance of the genotype based on MIC (resistance), liquid or solid medium (medium), whether the population was exposed to low (0.02 μg mL^−1^) or high (0.04 μg mL^−1^) antibiotic concentrations (CTX), and the time (24 and 48) postinoculation (time). The full factorial model gave an Akaike information criterion (AIC) value of 196.06, whereas a model with only two-way interactions resulted in an AIC value of 201.62. To further improve the model with two-way interactions, we dropped the two insignificant factors from this model (time and the CTX:Time interaction), resulting in a slight improvement in model support (AIC = 199.28). Both the Resistance:Medium and Medium:Time interactions were highly significant, and so we combined these into a single three-way interaction, resulting in a final model with appreciably better support than the full factorial model (AIC = 188.78). The final model is therefore resistance + medium + Resistance:CTX + CTX:Medium + Resistance:Time + Resistance:Medium:Time. Download Table S1, DOCX file, 0.02 MB. Copyright © 2023 Farr et al. 2023 Farr et al. https://creativecommons.org/licenses/by/4.0/ This content is distributed under the terms of the Creative Commons Attribution 4.0 International license.

### Fitness stratification in liquid medium is explained by periplasmic CTX concentrations.

Strains expressing mutation G238S had strikingly similar fitness measures when competed in liquid media, resulting in a stratification of the presented fitness landscape. This stratification is explained by a process that reduces the concentration of CTX within cells that express a G238S mutation. This process is distinct from the extracellular degradation of CTX that may occur through spontaneous (37) or catalytic hydrolysis by β-lactamase activity (38), which would reduce the available concentration of CTX to G238S and non-G238S genotypes, resulting in reduced fitness differences across all genotypes. Rather, sufficiently high activity by TEM-1 enzymes in the periplasmic space may reduce the periplasmic CTX concentration to subinhibitory concentrations, thereby reducing the concentration of CTX at penicillin binding protein (PBP) targets.

A model was devised which explains how CTX may select against non-G238S types—while causing a similar selection of G238S-expressing types—by modeling the reduction of steady-state CTX concentrations by TEM-1 activity within the periplasmic space. This model assumes that CTX quickly diffuses across the outer membrane of E. coli, with estimates of the time until half the external concentration has diffused into the periplasm being ~0.5 s (39, 40). While inside the periplasm, CTX is able to bind PBPs, ultimately affecting fitness (an assumption made in other kinetic models [41]). Both the rate of influx into the periplasm and the rate of binding of CTX PBPs can be assumed to be similar between mutants of low and high resistance. Consequently, the final steady state of periplasmic CTX is primarily determined by the rate (*R*) at which differing TEM-1 mutants reduce periplasmic concentrations of CTX. As such,

(1) $$a_{p(\text{steady})\;}=\;\frac{a}{R}$$

where $a_{p(\text{steady})\;}$ is the steady-state periplasmic concentration of CTX, $a_{\;}$ is the external concentration of CTX, and *R* is the factor by which the periplasmic concentration of CTX is lower than external concentrations. *R* was estimated using the MIC of mutants. We reasoned that the wild-type (WT) strain without TEM-1 has an $a_{p(\text{steady})\;}$ identical to that of the external environment (i.e., *R* is 1 for a mutant without TEM-1). Strains which express TEM-1 are able to reduce the concentration of CTX within the periplasm and the $a_{p(\text{steady})\;}$ at which growth becomes affected must be caused by higher extracellular CTX concentrations. Specifically, the MIC of a strain is the extracellular concentration at which $a_{p(\text{steady})\;}$ is equivalent to the MIC of an otherwise identical strain without TEM-1. From this, it follows that

(2) $$R=\;\frac{\text{MIC}}{{\text{MIC}}_{\text{WT}}{}_{}}$$

**FIG 6:**
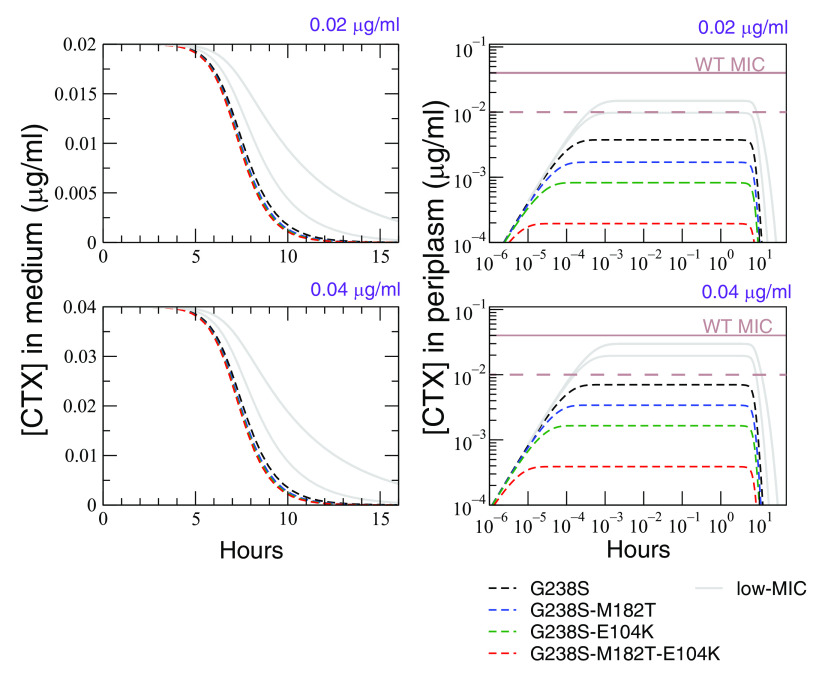
Simulation of the degradation of CTX in the medium and periplasmic space. The left two graphs present numerical simulations of the decrease in CTX concentration in the extracellular liquid medium of monocultures of the eight different TEM-1 mutants, inoculated into either 0.02 μg mL^−1^ (top) or 0.04 μg mL^−1^ (bottom). The predicted rate of degradation of CTX is based on assumptions of constant growth of the cells, an inoculum of 1× 10^5^ cells mL^−1^, estimates of TEM-1 enzyme concentration per cell, and the rate at which those enzymes hydrolyze CTX (see “Models of the degradation of CTX” for references to model and original data). The right two graphs present numerical simulations of the decrease in CTX concentration in the periplasmic space of the eight mutants tested in this study (again, at initial CTX medium concentrations of either 0.02 or 0.04 μg mL^−1^). This rate of degradation assumes constant diffusion of CTX into the periplasm across the genotypes and a rate of degradation determined by the level of resistance of each genotype. Gray lines represent the four low-resistance types in this study (lacking mutation G238S) and the four dashed/colored lines the four high-resistance genotypes carrying mutation G238S. The solid brown line represents the MIC of the WT strain without TEM-1, with cellular growth becoming inhibited when it reaches this periplasmic CTX concentration. The dashed brown line represents an estimation—derived from fitness assay data—of the proposed threshold periplasmic CTX concentration above which fitness is significantly affected. Note that some of the curves almost coincide since the estimated parameters used in the model are nearly identical.

**FIG 7:**
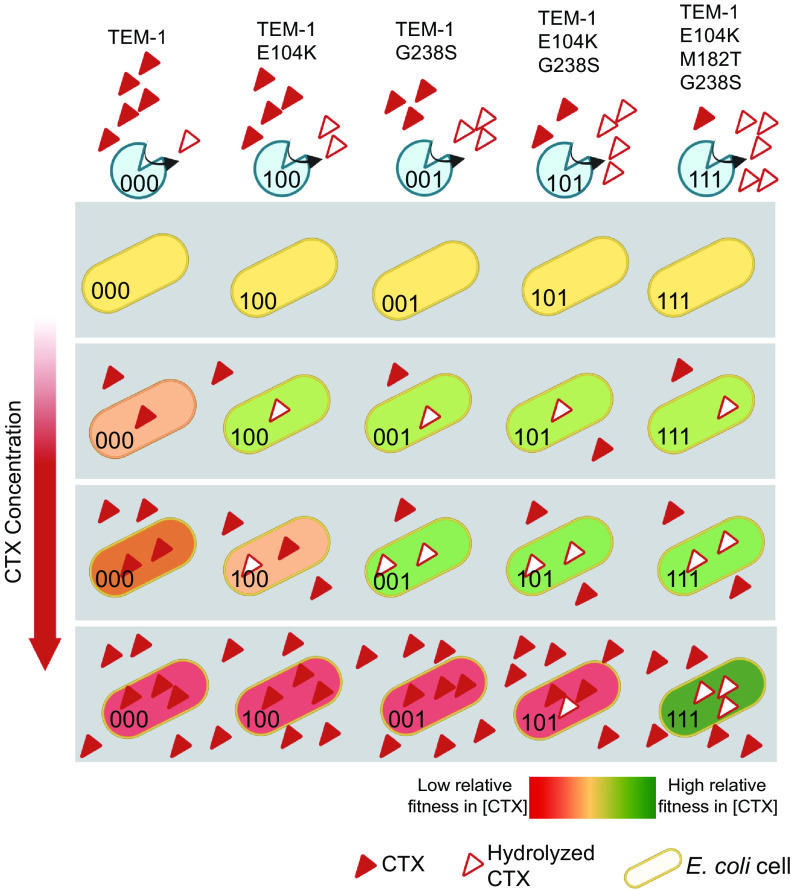
Proposed cause of equivalent fitness among competing genotypes at sublethal antibiotic concentrations. Presented are the relative fitnesses of five representative TEM-1 mutant genotypes competing with each other in particular concentrations of CTX (signified by a single gray box). At low and intermediate initial concentrations of CTX, more active TEM-1 mutants are able to hydrolyze CTX, effectively reducing the periplasmic concentrations to subinhibitory levels (see Fig. 6) and allowing fitness to be maximal. This internal hydrolysis of CTX results in classes of types with equivalent fitnesses, while susceptible mutants with higher concentrations of CTX in the periplasm have lower fitness. As the concentration of CTX in the medium increases (see lower gray boxes), this class of fitness-equivalent mutants is predicted to decrease in number, leaving only those which can degrade internal CTX to sufficiently low concentrations. Note that this figure presents the fate of cells at early stages of competition, before cells have a chance to alter the environmental concentrations of CTX. As the density of variants that can hydrolyze periplasmic CTX increases due to growth, eventually the environmental CTX will be degraded, removing the relative fitness difference of resistant and susceptible cells. If the density of high-resistance variants increases rapidly and leads to a rapid degradation of environmental CTX, the time window in which differences in TEM-1 activity against CTX for low-resistance variants (i.e., TEM-1 versus TEM-1 E104K) impact fitness may be small, leading to fitness stratification for the low-resistance types.

Combining equations 1 and 2, the steady-state periplasmic concentration can be calculated for any of our TEM-1 mutants. A detailed explanation of this model is found in supplemental files (35). Figure 6 presents numerical simulations of this model and how quickly $a_{p(\text{steady})\;}$ is reached compared with effects on CTX concentration in the extracellular medium as a result of TEM-1 activity. While the mutants require at least 7 h until the extracellular concentration is reduced by half, our model predicts that $a_{p(\text{steady})\;}$ is arrived at within seconds. The four G238S encoding mutants have various concentrations of $a_{p(\text{steady})}$ (see right-hand panels of Fig. 4), but there appears to be a critical periplasmic concentration of CTX below which growth proceeds largely unaffected. By observing the selective CTX concentrations which distinguish the E104K from G238S mutants in fitness assays (which occurs at external CTX concentrations of ~0.02 μg mL^−1^ in liquid medium), this critical periplasmic concentration was determined to be approximately 0.01 μg mL^−1^. The maintenance of periplasmic CTX concentrations below this critical value by G238S mutants allows these types to compete as though the environment was free of CTX (see Fig. 7 for a cartoon of this process) and explains the observed stratification of fitness.

## DISCUSSION

Here, a new approach for investigating empirical fitness landscapes is reported: assaying the fitness of each genotype in the landscape by direct competitions with its local mutational neighborhood. This approach is a useful advance in the empirical analysis of fitness landscapes, especially for simulating strong clonal interference (42, 43) or conditions invoking nontransitive fitness interactions (20, 44). Nontransitive interactions between genotypes could be expected if the β-lactamase mutants significantly reduced the environmental CTX concentration, such as in spatially structured environments with lower CTX concentrations. While our approach should readily identify nontransitive interactions that lead to the deformation of the landscape, no such interactions were observed. Instead, regardless of the number of competitors, fitness was remarkably binary in the presence of antibiotics: genotypes had either high or low fitness, depending on the presence of large-effect mutation G238S. This strong dependence on a single mutation and its resulting stratification of the landscape may explain why more subtle interactions remained undetected. We expect that for landscapes involving a stronger influence of different genotypes on the competitive conditions (e.g., environmental antibiotic concentration), such as when the β-lactamases were expressed via a multicopy plasmid with a stronger gene dosage, these interactions exist and can be unveiled using similar approaches.

The fitness landscapes presented here showed not a single-genotype adaptive “peak” but rather a “stratification” of genotypes with equivalent fitness under the tested conditions. In both structured and unstructured environments, high-resistance genotypes had similar fitnesses at subinhibitory concentrations of CTX. In our eight-mutant fitness landscape, equivalence of the fitness of competing genotypes was determined by the presence of the G238S mutation. This similarity in fitness among high-resistance types is partially explained by the location and activity of β-lactamase in the periplasmic space, where it can reduce the local concentration of CTX (Fig. 6) and prevent inhibitory concentrations of CTX binding to target PBPs (45). Provided sufficient enzymatic hydrolysis, we predict that the concentration of CTX in the periplasm is brought below a threshold inhibitory concentration within seconds, resulting in equivalent fitnesses of types with similar relative fitnesses in the absence of CTX. The rapid degradation of CTX in the periplasm can help explain the observed stratification, which appears extreme relative to the mild stratification observed in fitness landscapes of *gyrA* mutants, which ameliorate the effects of nalidixic acid by avoiding target binding (46).

While our hypothesis is consistent with the observed fitness stratification, the actual molecular causes of this stratification remain uncertain. The stratification exists despite significant differences in β-lactamase functionality, and hence resistance level, among high-resistance genotypes. We can speculate that either the target of CTX—the PBPs—are functionally robust to low periplasmic concentrations of CTX or mildly lowered PBP function does not affect rates of cellular growth. Nonlinearities in the relationship of function and fitness are common and have, for example, been observed in mutants of isopropylmalate dehydrogenase (7). Such nonlinearities often reflect constraints from other parts of metabolism which determine fitness together with the target function (45), which reduces the sensitivity of fitness to perturbation in any one function (46). In our relatively simple system, the robustness of cell fitness to concentrations of periplasmic CTX below the threshold is complicated by the production of nascent PBPs during growth (47), the multiplicity of PBPs which bind CTX (48), and the involvement of other enzymes controlling cell wall synthesis and degradation (49). We hope that future research will elucidate the molecular causes of the insensitivity of cells to these low concentrations of CTX, a phenomenon we predict to be found in other resistance systems that degrade antibiotics.

Our observation of fitness stratification may help refine our understanding of how sub-MICs of antibiotic, as found in environmental settings such as wastewater (31, 50–53), may select for antibiotic-resistant strains (14, 32, 54–57). While it is clear that antibiotic-resistant genes confer a benefit under nonlethal conditions (14, 32), little is known about the evolution of such genes under these conditions. This study provides grounds to doubt that sub-MIC environmental concentrations of antibiotics may select for mutations conferring the highest resistance, an idea that has recently found support in experiments with the carbapenemase OXA-48 (58). If many genotypes have equivalently high fitness above a threshold value of resistance, there are many factors that could determine which variants eventually predominate. For example, mutation bias (59, 60), trade-offs between resistance and growth (61), and collateral sensitivity to other antibiotics (62, 63) could be important. Selection for resistant types in natural environments may be further complicated by the striking inverse relationship between resistance and fitness in spatially structured sub-MIC environments, a phenomenon which is associated with filamentation of susceptible types as previously reported (26). It is clear that a degree of environmental spatial structuring is common to microbial populations in their natural habitats. It is thus relevant to consider when environmental spatial structuring and selection at subinhibitory antibiotic concentrations may select for less resistant genotypes.

The lack of selective benefit for genotypes such as TEM-52 in our study conflicts with the wide and frequent isolation of this mutant β-lactamase in both environmental (64) and clinical (65) settings. A scenario accounting for the global distribution of TEM-52 that is compatible with our observations is one in which (i) environmental conditions with sublethal antibiotic concentrations select for limited resistance, (ii) upon migration of the β-lactamase hosts to clinical environments, or other rare habitats with high antibiotic concentrations, there will be relatively short bouts of selection for high-resistance mutations, and (iii) these variants are then maintained under the environmental conditions, where all high-resistance alleles have equivalent fitnesses and near neutral evolution predominates.

There are important considerations to make when applying these findings and ideas to real-world scenarios. For practical reasons, we used equal starting frequencies of the competitors and can only speculate how combining competitors at extreme frequencies—such as when mutations arise *de-novo*—would affect resulting fitness differences. It is possible that the fitness benefits of genotypes are independent of starting frequency, resulting in the eventual fixation of genotypes. Alternatively, there may be negative-frequency-dependent interactions which establish an equilibrium of high- and low-resistance types as a result of CTX degradation and fitness resistance trade-offs (22). It is also unclear whether the spatial-structure-associated benefit of low-resistance genotypes is caused only by β-lactams such as CTX (this study) and carbenicillin (26) or whether this phenomenon extends to other antibiotics which cause milder changes in the cellular aspect ratio of sensitive strains (66). An improved understanding of the relationship between fitness and resistance across resistance mechanisms will provide a fuller picture of how clinically relevant strains evolve and depend on specific selective conditions.

## MATERIALS AND METHODS

### Strains, media, and growth conditions.

All strains used in this study derive from Escherichia coli MG1655 (Table 1). E. coli MG1655 *galk*::*SYFP2-cat* (DA28100) and E. coli MG1655 *galk*::*mTagBFP2-cat* (DA28102) were kindly gifted by Peter A. Lind (Uppsala University, Uppsala, Sweden). All experiments were performed in minimal medium composed of the following: 8.5 g L^−1^ of Na_2_HPO_4_·2H_2_O, 3 g L^−1^ of KH_2_PO_4_, 0.5 g L^−1^ of NaCl, 1 g L^−1^ of NH_4_Cl, 4.93 g L^−1^ of MgSO_4_·7H_2_O, 147 mg L^−1^ of CaCl_2_·2H_2_O, 0.2% (wt/vol) Casamino Acids (Difco), 2 mg L^−1^ of uracil, 1 μg L^−1^ of thiamine, and 0.4% (wt/vol) glucose. Agar was added at 1.5% (wt/vol) in order to make solid medium and was autoclaved separately from other components. Expression of β-lactamase was induced by addition of 50 μM isopropyl β-d-1-thiogalactopyranoside (IPTG) to the competition medium. CTX solutions were prepared from cefotaxime sodium salt (Sigma), diluted in minimal medium solution and stored as stock solution at 5.12 mg mL^−1^ at −20°C before final dilution to stated working concentrations. Strains were prepared for flow cytometry by dilution in M9 salt solution (8.5 g L^−1^ of Na_2_HPO_4_·2H_2_O, 3 g L^−1^ of KH_2_PO_4_, 0.5 g L^−1^ of NaCl, 1 g L^−1^ of NH_4_Cl, 4.93 g L^−1^ of MgSO_4_·7H_2_O) that was filtered using a 0.2-μm syringe filter. Strains were grown at 37°C. Liquid cultures were always aerated with orbital shaking.

### Strain reconstruction.

All TEM-1 alleles in this study were reconstructed into a clonal strain of Escherichia coli MG1655 featuring a fluorescent *bfp* reporter gene inserted into *galK* (*galK*::mTagBFP2-FRT) (32). TEM-1 alleles—derived from a previous study (5)—were amplified by PCR and inserted into a remaining section of *galK* using a quick and easy E. coli gene deletion kit (Gene Bridges), using ampicillin resistance conferred by the TEM-1 genes to select for double recombinants. Individual ampicillin-resistant colonies were isolated, cryogenically stored, and amplicon sequenced to confirm the absence of additional mutations in TEM-1. Each biological replicate used in subsequent assays represents an individual transformant.

### MIC measurements.

All MIC assays of the TEM-1 mutants were performed in the identical minimal medium used for competitions, using an inoculum similar to that used in experimental competitions in liquid medium. Assays were initiated with overnight cultures of each biological replicate in minimal medium, grown for approximately 18 h until stationary phase. Cultures were then subcultured with a 1:100 dilution and grown for ~3 h to a final cell density of 1.5 × 10^8^ mL^−1^. Meanwhile, 2-mL deep-well 96-well plates were filled with 400 μL of minimal medium featuring IPTG and a 2-fold dilution series of CTX over 12 rows (25.6 to 0.0125 μg mL^−1^ of CTX, except for one row intended for the E104K M182T G238S genotype which ranged from 102.4 to 0.05 μg mL^−1^ of CTX). A further 1:10 dilution of the subculture was made was made and 3 μL of each genotype was added to the wells of each column, resulting in final concentration of cells of 1.5 × 10^5^/mL in each well. Plates were sealed with Breathe-Easier sealing film (Diversified Biotech) and shaken with orbital shaking at 750 rpm for 24 h, whereupon 200 μL from each well was transferred to transparent 96-well plates for measurement of optical density at 600 nm (OD_600_) by a Victor3 microtiter plate reader (PerkinElmer). MICs were interpreted as the lowest concentration of CTX that maintained cultures at an OD_600_ of less than 0.1. Determination of the MICs of DA28200 and DA28202 was performed similarly to the description above, except that assays were initiated from overnights cultures, diluted into 200-μL volumes of M9 medium with CTX (in 96-well plates), and inhibitory concentrations were assessed visually. MIC values of individual genotypes are reported as median values, while MIC values of groups of genotypes are the means of each genotype’s median MIC values.

### Pairwise fitness assays.

Pairwise fitness assays involved direct competition of each stated genotype with a common *yfp*-expressing competitor (E. coli MG1655 *galK*::SYFP2-FRT), performed either in liquid or on solid medium. Liquid medium competitions were initiated with overnight monocultures of each competitor grown in 2 mL of minimal medium. Overnight cultures were grown for ~18 h, and then each competitor was subcultured together with a 1:200 dilution into fresh minimal medium and incubated for ~3 h. Meanwhile, the competition medium was prepared: 794 μL of minimal medium containing IPTG and specified concentrations of CTX (see Results) were aliquoted into rows of a 2-mL deep-well 96-well plate. Subcultures were then added to the competition medium, by pipetting 6 μL of the subculture into the competition medium. To measure the initial ratio and concentration of cells, 50 μL was sampled from competition wells without CTX to avoid any possible effect of CTX on the ratio of each competitor. These samples were each added to 200 μL of filtered M9 salt solution inside the wells of a 96-well plate, and BFP^+^ and YFP^+^ cellular events were quantified by flow cytometry using a Macsquant10 (Miltenyi Biotec). The competition plates were then sealed with Breathe-Easier sealing film (Diversified Biotech) and incubated at 37°C with orbital shaking at 750 rpm. After 24 and 48 h of competition, 10-μL samples were taken from each well and diluted 1:500 in M9-salt solution to allow flow cytometry of the ratio and concentration of the competitors.

Flow cytometry was performed using a medium flow rate using SSC 1.4 as a trigger (no secondary trigger) with 25,000 events counted per sample. All events measured as >1 by the 450/50-nm and 525/50-nm channels were respectively counted as BFP^+^ and YFP^+^ events. Fitness assays performed on solid medium were performed similarly to the liquid-medium competitions with the following distinctions. Overnight cultures of each competitor were subcultured together with a 1:1,000 dilution into fresh minimal medium, incubated with shaking for 3 h, and then further diluted (1:10). Samples of this final dilution were taken for cellular quantification by flow cytometry. A drop of 1 μL of the mixed culture was spotted onto minimal medium solidified with agar (containing a relevant concentration of CTX). Spotting was performed twice to allow the destructive harvesting of colonies at 24 and 48 h. Harvesting was performed by scooping each colony into the base of a sterile 1-mL pipette tip, with the cells resuspended by vortexing the pipette tip in a microcentrifuge tube containing 1 mL of M9 salt solution. The cell solution was then diluted 1:100 in M9 salt solution and the ratio and concentration of cells were quantified by flow cytometry. Selection coefficients were calculated as *s* = [ln(*R*(*t*)/R(0))]/[*G*] (where *R* is the ratio of a genotype relative to competitors at time [*t*] and *G* is the number of generations) (67). The number of generations was calculated by ln(final population/initial population)/ln(2). Presented selection coefficient values for the genotypes were baseline adjusted to make the selective coefficient for TEM-1 equivalent to 0. All pairwise fitness assays were performed with three biological replicates each on a separate occasion.

### Four-genotype fitness assays.

We measured the relative fitness of the four genotypes connected by a single mutation to each node of the TEM-1 to TEM-52 fitness landscape, as well as the relative fitness of all eight genotypes competed together. Competitions were performed in liquid or on solid medium supplemented with either 0, 0.02, or 0.04 μg mL^−1^ of CTX. For each replicated assay, all eight strains were cultured for ~18 h overnight in 2 mL of minimal medium. The strains were then mixed by adding 2 μL of each culture in a 1:1:1:1 ratio into 2 mL of minimal medium (or into 4 mL in the case of the eight genotype competitions). Strains were then grown together for 3 h and then diluted 1:10 in M9 salt solution, and the total cellular number of BFP^+^ events was measured by flow cytometry as in the pairwise fitness assays (see above). Subcultures were then inoculated into the competitive media. Liquid medium competitions were initiated with 2.4 μL of each mixed subculture inoculated into 2 mL of minimal medium supplemented with IPTG and either 0, 0.02, or 0.04 μg mL^−1^ CTX. Solid medium competitions were then initiated with a 1-μL drop of subculture spotted onto minimal medium supplemented as in the liquid competitions. Competitions were founded with approximately 3 × 10^4^ cells mL^−1^ for liquid competitions and ~3 × 10^4^ cells in the case of spatially structured competitions. The concentration of BFP^+^ cells in the subculture was further quantified by flow cytometry, and the mean values of these measures—before and after inoculation in the test media—were used as measures of the initial concentration of cells in each competition. A sample of 1 mL of subculture was then added to a cryogenic tube supplemented with glycerol and flash frozen for subsequent amplicon sequencing (see below). Liquid and solid medium competitions were then incubated at 37°C (with shaking for the liquid cultures) for 24 or 48 h until sampling. Sampling of liquid cultures was performed by removing 150 μL of competition medium after 24 and 48 h. Glycerol was added to the sample, which was then frozen at −70°C for amplicon sequencing at a later date. An additional 10-μL sample of competition medium was taken, diluted 1:400, and used to measure the concentration of BFP^+^ cells by flow cytometry (see details for pairwise competition methods for flow cytometry). Colonies that had grown in solid medium after 24 and 48 h were destructively harvested in the same way as for the pairwise competitions (see above). Colonies were resuspended in 1 mL of M9 salt solution and the samples stored and BFP^+^ events quantified in the same fashion as the liquid medium samples. A total of four biological replicates of each genotype was used in this experiment, with a different biological replicate competed on separate occasions and competition medium freshly prepared for each replicate.

Simultaneously with these competitions, controls experiments were conducted on isogenic populations of each of the eight competitors. Instead of mixing each competitor with 3 other genotypes, isogenic subcultures were made that were otherwise treated identically to samples from four-genotype competitions. Flow cytometry measures were then taken to establish the degree of filamentation in each population subjected to CTX. To do so, these control populations were measured after 24 and 48 h using the FSC channel of a Macsquant10 instrument (Miltenyi Biotec). Flow cytometry was performed with the same setting as used in pairwise fitness assays (see above), including gating of events >1 in the 450/50-nm channel to remove nonfluorescent particles. The arbitrary FSC value that defined the largest 1% of events in populations in CTX-free environments was established, and the percentage of samples that exceeded that FSC value was established for populations subjected to CTX. Three biological replicates were conducted for each genotype and treatment.

### Amplicon sequencing and data analysis.

Amplicon sequencing was used to measure the relative frequency of genotypes in the four- and eight-genotype competitions to allow calculations of relative fitness. Primers were designed to produce a 475-bp amplicon which spanned the three mutation sites. Forward and reverse primers were each labeled with a 5′ 4-bp sequence to allow multiplexing. Each of the eight four-genotype competitions were amplified with a unique pair of indices for each treatment and each replicate (Table S2). Frozen samples from each time point were thawed and used directly as a template for PCR. Different volumes of sample were used as the template from the 24- and 48-h time points (1 μL) compared to intimal time points (2 μL). PCRs were performed on each occasion with the template of a different replicate. Reactions were performed in a total volume of approximately 30 μL, including 15 μL of Phusion Flash high-fidelity PCR master mix (Thermo Scientific), forward and reverse PCR primers to a final concentration of 0.5 μM, and the thawed template. PCR was performed over 30 cycles with an initial 5-min incubation step to allow for cell lysis. Amplicons from each reaction were confirmed by electrophoresis. PCR products with differing indices from each treatment and replicate were then pooled and purified with NucleoSpin gel and PCR cleanup (BIOKE). The TruSeq library preparation kit (Illumina) was then used to prepare libraries for MiSeq PE-250 sequencing (Illumina), with a preparation done for each pool of nine samples. Library preparation and high-throughput sequencing were performed by the Cologne Center for Genomics.

10.1128/mbio.00098-23.8 TABLE S2 Primers used during amplicon sequencing. Download Table S2, DOCX file, 0.02 MB. Copyright © 2023 Farr et al. 2023 Farr et al. https://creativecommons.org/licenses/by/4.0/ This content is distributed under the terms of the Creative Commons Attribution 4.0 International license.

The sequencing data were analyzed with CLC Genomics Workbench 11.0. We trimmed the sequences with Trims Reads 2.1, with standard settings except for a quality limit of 0.001 (equivalent to a Phred score of 30) and minimum number of nucleotides of 260. All broken pairs were discarded at this step. Next, we mapped the trimmed reads to a set of reference sequences, representing all possible combinations of our custom barcodes and TEM alleles (i.e., not only those combinations that were expected but all possible permutations). For this step, Map Reads to Reference was used with standard settings, except a length fraction of 0.995 and similarity fraction of 0.995 (i.e., the reads have to be perfect matches to the reference to map). We then used the total read count mapped to each sequence as the estimate of that sequence’s frequency in the population. (Note that as an alternative approach, we first subdivided the data from each Illumina library based on our custom barcodes for different conditions and trimmed the barcodes, using Demultiplex Reads 1.0, and then trimmed and map these reads as above but to reference sequences without the custom primers present. The resulting frequencies of alleles were nearly identical).

We found unexpected combinations between TEM alleles and our custom barcodes at appreciable frequencies, with the three possible sequences with a single unexpected position in the TEM sequence being more common (mean frequency ± standard deviation = 0.023 ± 0.019, determined over all nodes in the fitness landscape) than the sequence with two unexpected positions in the TEM sequence (0.003 ± 0.002). These unexpected alleles therefore appear to have arisen due to recombination in the first PCR step. We know which real alleles were in the inoculum, and each allele other than the “nodal progenitor” (i.e., TEM-1 for the competition between TEM-1 and all three one-step mutants) has a unique mutation that allows us to determine its frequency from the number of (essentially full length) sequencing reads that mapped to each of the eight TEM alleles. The frequency of the nodal progenitor is then one minus the sum of the three other genotypes. If the estimated frequency of the nodal progenitor was very low (<0.001), we simply used the relative frequency of reads that mapped to the nodal type as an approximation of its frequency. Genotype frequencies were then converted into selection coefficient values using the same method as described in “Pairwise fitness assays” above.

### Models of the degradation of CTX.

A full explanation of the model describing the degradation in the periplasmic and extracellular CTX in monocultures is available in supplemental files (see “Modeling of antibiotic concentration reduction in periplasmic space” in reference 35). As detailed, simulations of the degradation of extracellular CTX are based on assumptions of doubling time, the starting inoculum, and estimates of TEM-1 enzyme concentration in the periplasm, done through determination of *R* in equation 2 (see “Modeling of antibiotic concentration reduction in periplasmic space” in reference 35). This model was then run on Fortran with output data available via Zenodo (35). Simulations for the periplasmic degradation of CTX are based on the MIC values found in Table 1, which were used to approximate the single-cell MICs of the strains (see the workbook file “Input data CTX degradation model” in supplemental files [35]). Single-cell MICs were used to limit the effect of the inoculum on the relative ability of individual cells to remove periplasmic CTX. These simulations assumed that the starting concentration of CTX in the periplasm was 0 μg mL^−1^ and increased by the estimated rate of diffusion into the periplasm until the steady state was reached (as calculated in the workbook file “Input data CTX degradation model” [35]). Simulations were again performed with Fortran.

### Data availability.

Raw sequence reads for the four-genotype competitions are available via NCBI BioProject number PRJNA844400 (68). Scripts used for the presentation of fitness data (Fig. 3 and Fig. S3) and for the analysis of flow cytometry data (Fig. 5A) are similarly available via Zenodo in the folder “Scripts for Figs3_5” (35). Supplemental data, including calculations of MIC measures (Fig. 1 and Table 1), selection coefficients of pairwise competitions (Fig. 2 and Fig. S1), selection coefficients of four-genotype competitions (Fig. 3 and 4 and Fig. S3), and filamentation measures (Fig. 5), are similarly available via Zenodo (35). All input files, output files, code, and notes concerning modeling of CTX degradation are similarly found in the zipped folder “Model notes, input data, code and output data for CTX degradation simulation in figure 6, zip” via Zenodo (35).
