# Supplementary figures and images for: The Fitness of Beta-Lactamase Mutants Depends Nonlinearly on Resistance Level at Sublethal Antibiotic Concentrations
Source: mBio. 2023 Apr 27;14(3):e00098-23. doi: 10.1128/mbio.00098-23 (PMC10294655; doi:10.1128/mbio.00098-23)

## Relative fitness of each genotype under altered concentration of CTX : liquid media

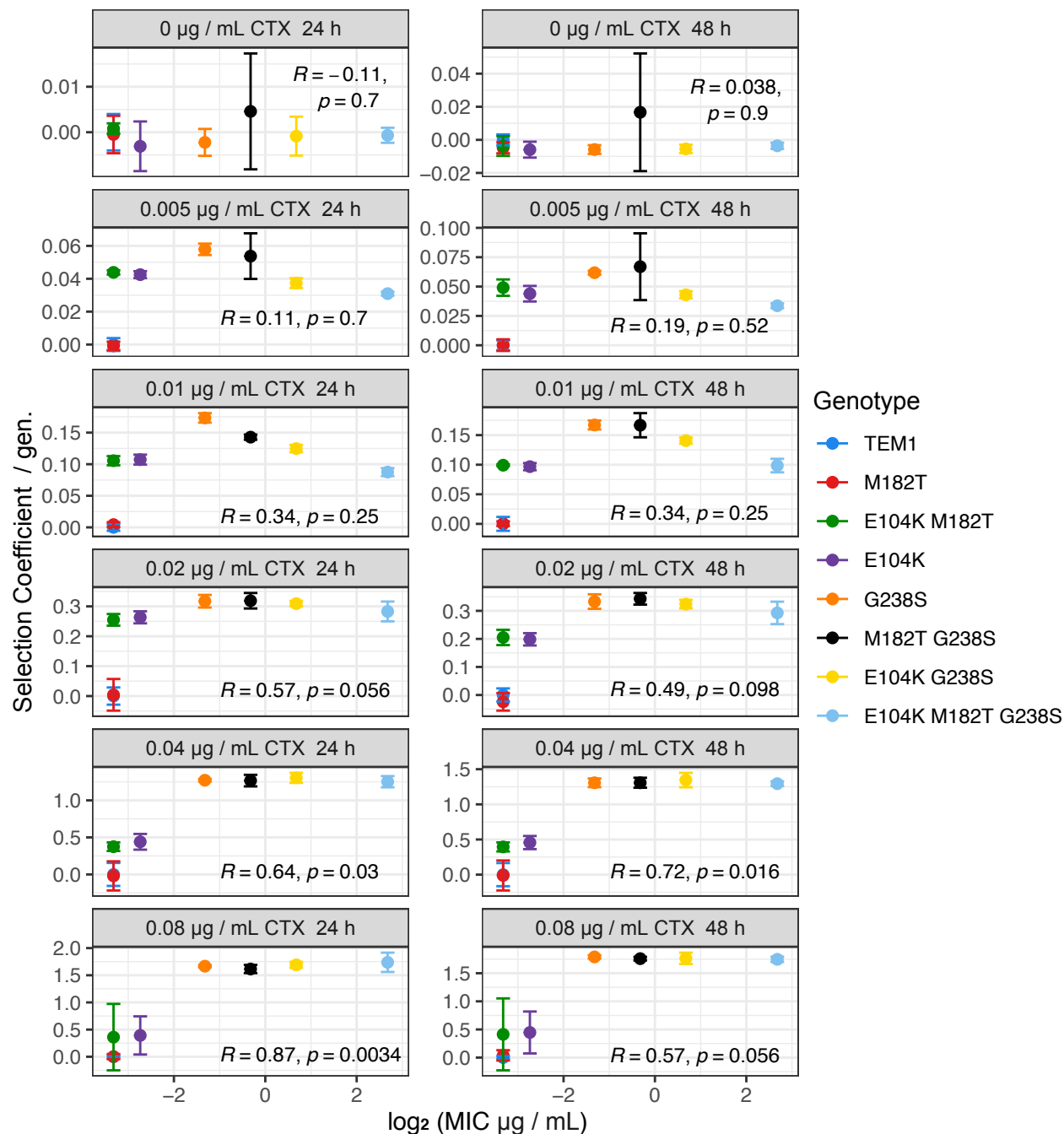

Supplement: FIG S1 [file mbio.00098-23-s0001.pdf]

## Relative fitness of each genotype under altered concentration of CTX : Solid media

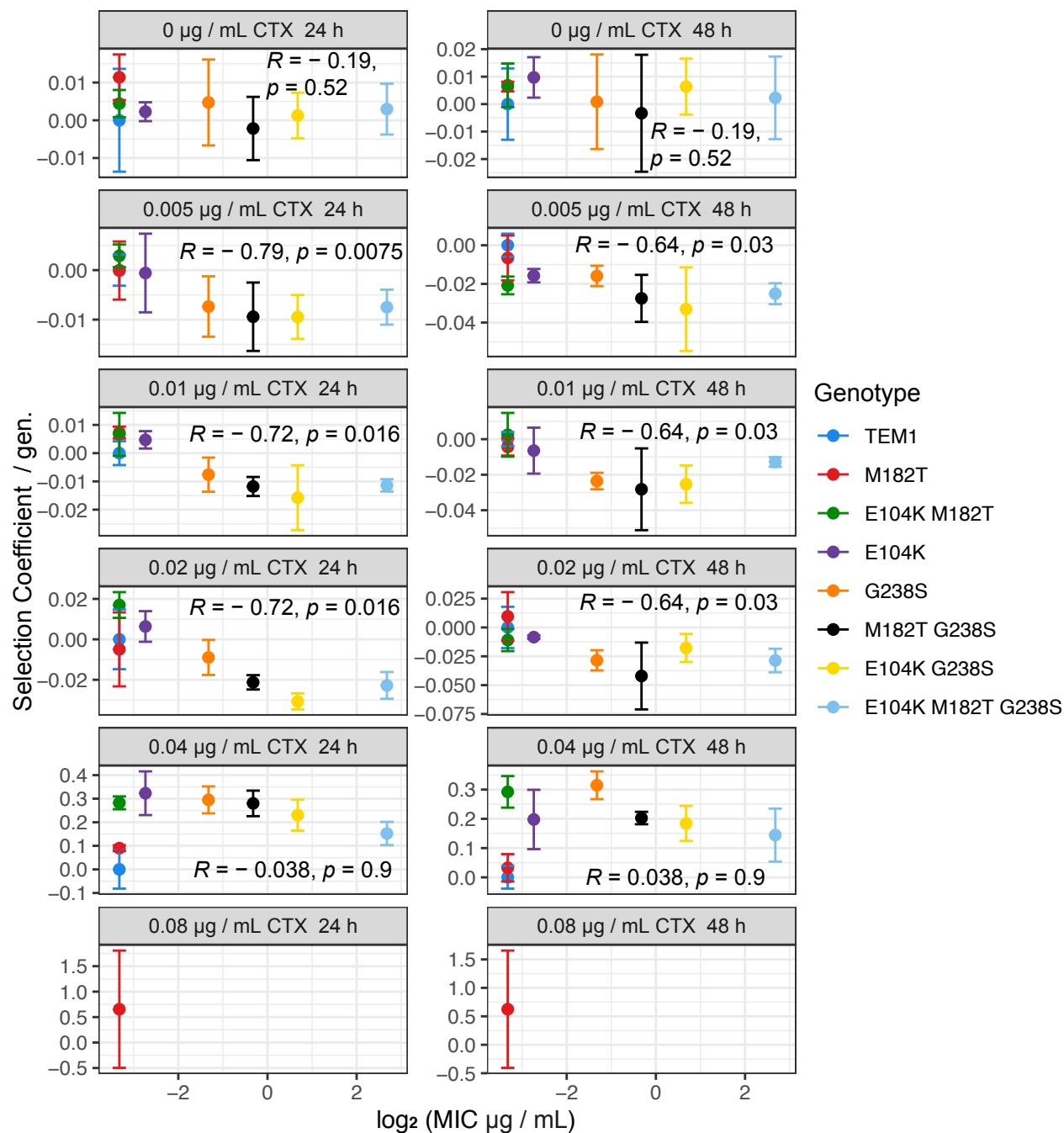

Supplement: FIG S2 [file mbio.00098-23-s0002.pdf]

## Relative fitness of each genotype under altered concentration of CTX : 24 hours

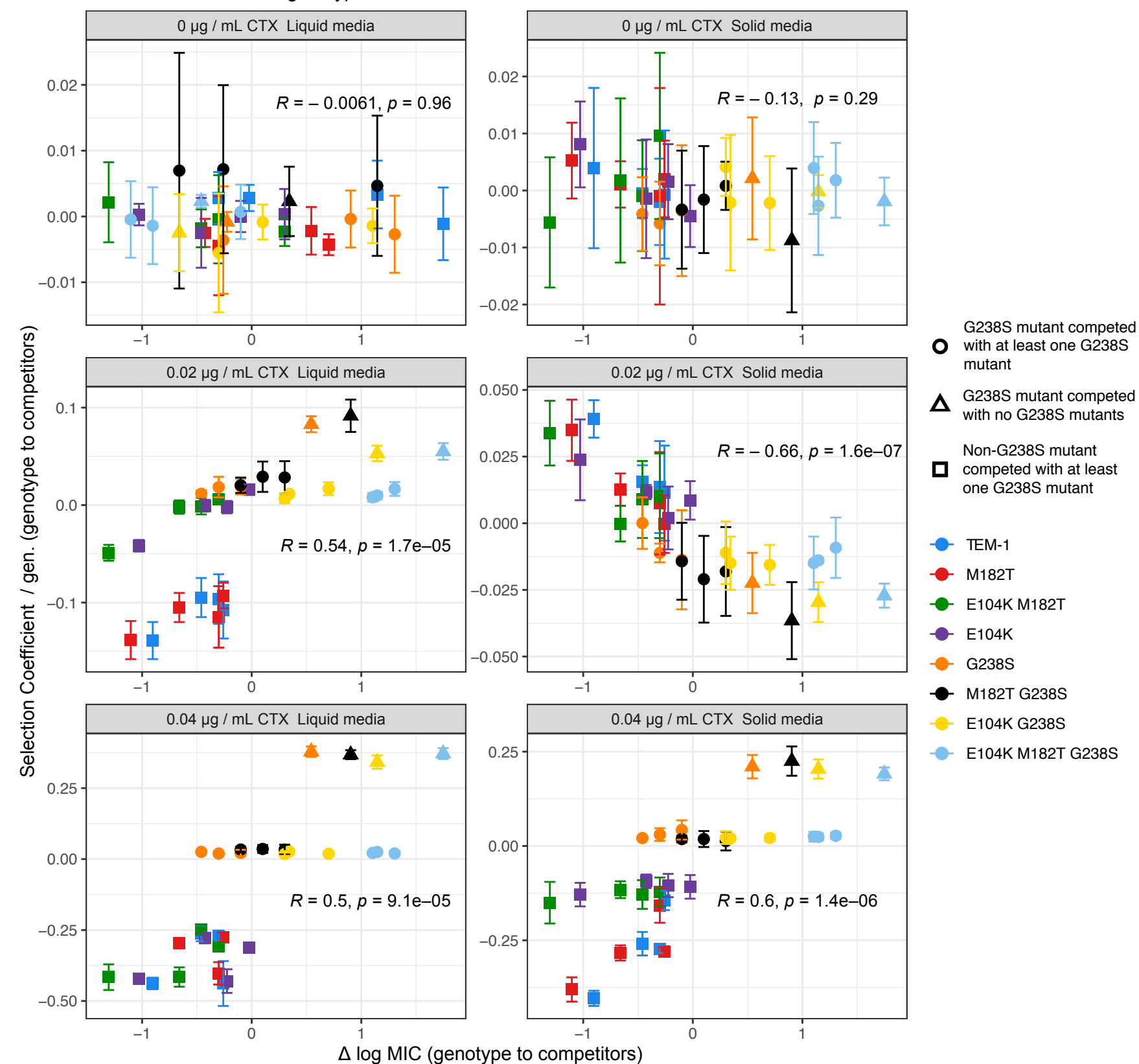

Supplement: FIG S4 [file mbio.00098-23-s0004.pdf]

## Relative fitness of each genotype under altered concentration of CTX : 24 hours

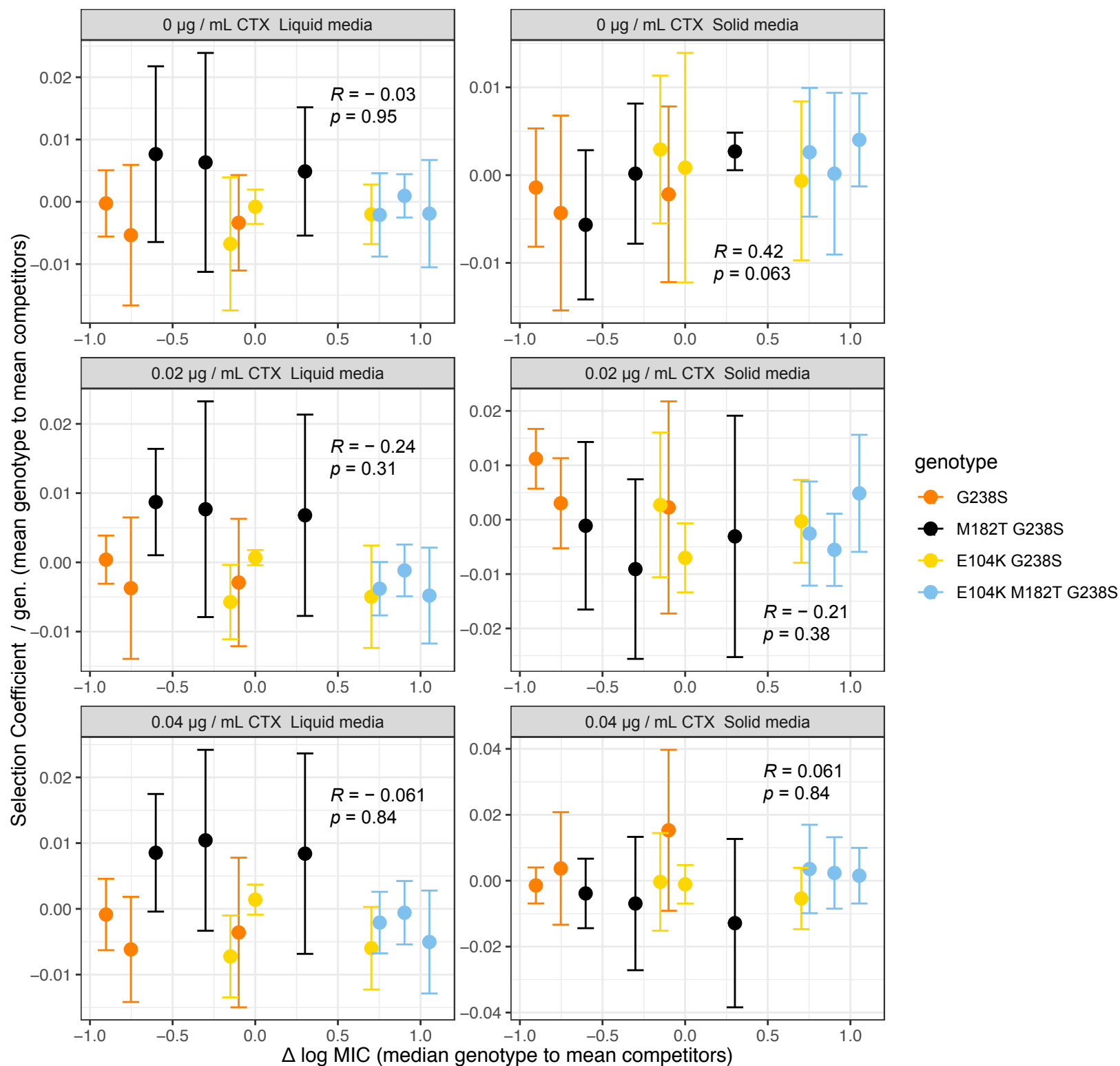

Supplement: FIG S5 [file mbio.00098-23-s0005.pdf]

Relative fitness of each genotype under altered concentration of CTX : 48 hours

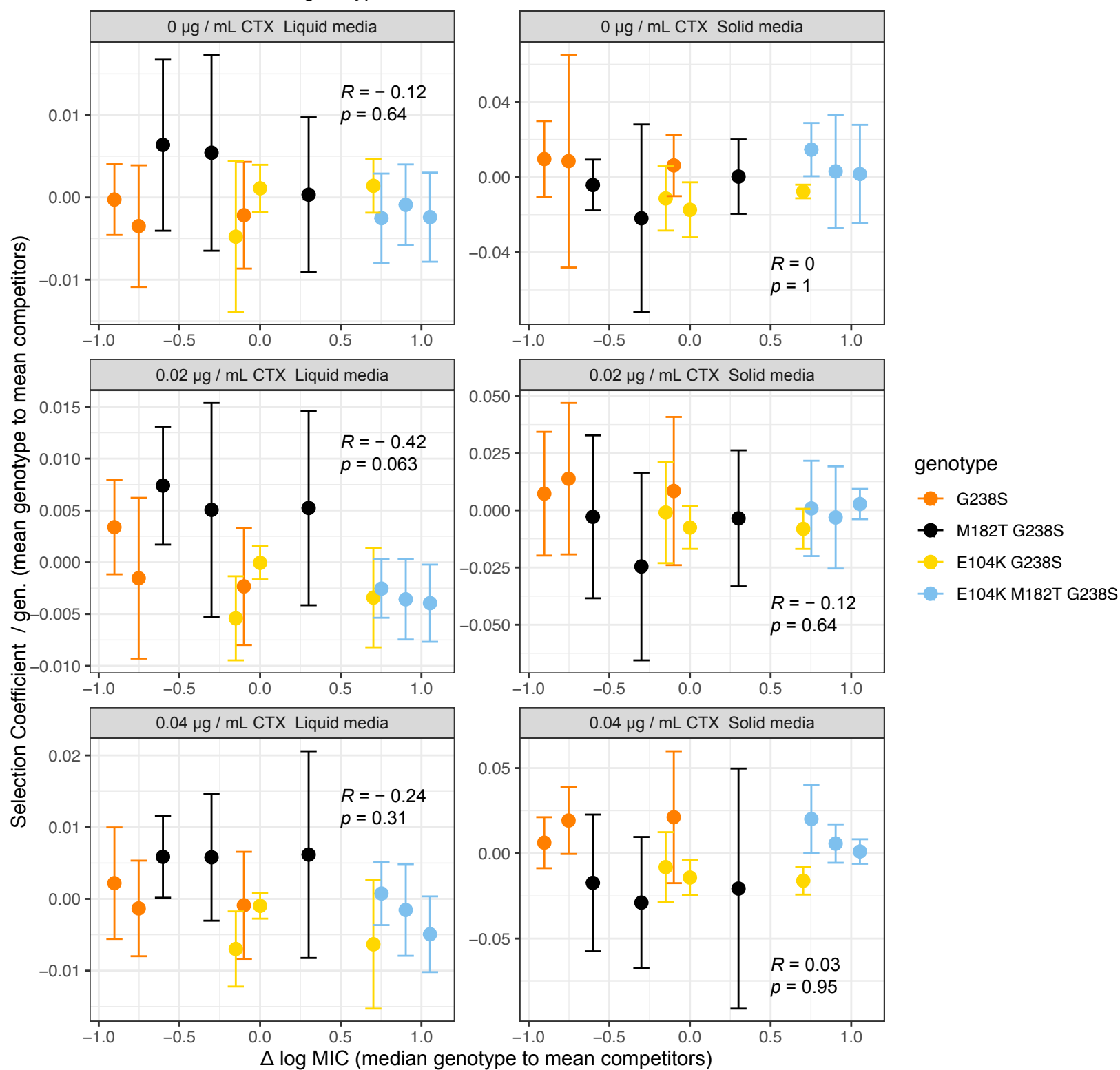

Supplement: FIG S6 [file mbio.00098-23-s0006.pdf]
