# Supplementary material for: The Fitness of Beta-Lactamase Mutants Depends Nonlinearly on Resistance Level at Sublethal Antibiotic Concentrations
Source: mBio. 2023 Apr 27;14(3):e00098-23. doi: 10.1128/mbio.00098-23 (PMC10294655; doi:10.1128/mbio.00098-23)
Supplement: FIG S3 [file mbio.00098-23-s0003.pdf]

Supp. Fig. S3

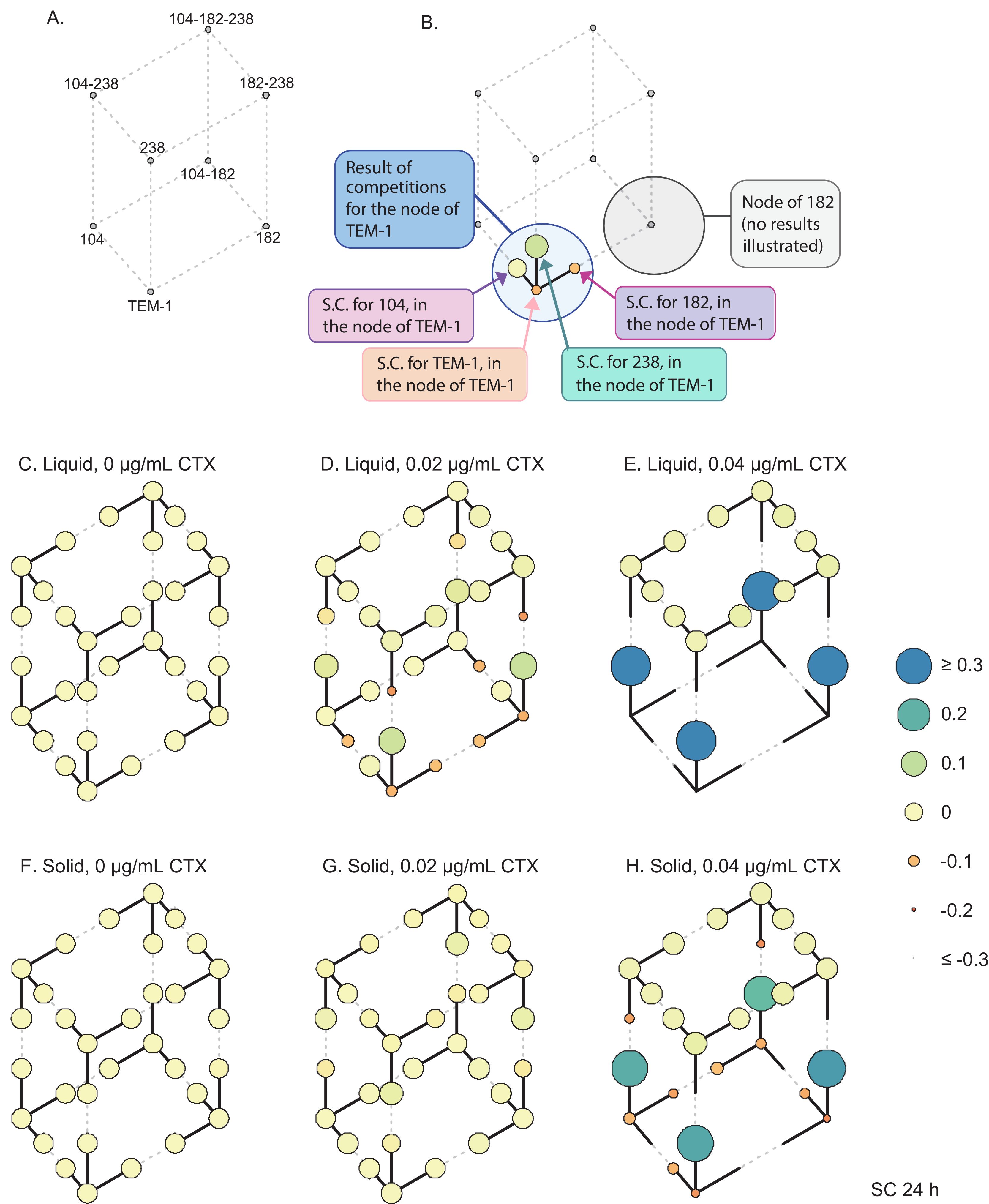

A.
104-182-238
104-238
182-238
238
104-182
104
182
TEM-1
B.
Result of competitions for the node of TEM-1
Node of 182 (no results illustrated)
S.C. for 104, in the node of TEM-1
S.C. for 182, in the node of TEM-1
S.C. for TEM-1, in the node of TEM-1
S.C. for 238, in the node of TEM-1
C. Liquid, 0 µg/mL CTX
D. Liquid, 0.02 µg/mL CTX
E. Liquid, 0.04 µg/mL CTX
F. Solid, 0 µg/mL CTX
G. Solid, 0.02 µg/mL CTX
H. Solid, 0.04 µg/mL CTX
≥ 0.3
0.2
0.1
0
-0.1
-0.2
≤ -0.3
SC 24 h
