# Supplementary material for: The Fitness of Beta-Lactamase Mutants Depends Nonlinearly on Resistance Level at Sublethal Antibiotic Concentrations
Source: mBio. 2023 Apr 27;14(3):e00098-23. doi: 10.1128/mbio.00098-23 (PMC10294655; doi:10.1128/mbio.00098-23)
Supplement: TABLE S1 [file mbio.00098-23-s0007.docx]

**Table S1: General linear model (GLM) for flow cytometry data.**

| Coefficient^a^ | Estimate ±SEM^b^ | *t* value | *P* value |
| --- | --- | --- | --- |
| Intercept | -2.817 ±0.181 | -10.995 | < 2 × 10^-16^ |
| Resistance | 0.098 ±0.044 | 2.284 | 0.028 |
| Medium | 0.558 ±0.154 | 3.613 | 3.90 × 10^-4^ |
| Resistance:CTX | 5.386 ±1.240 | 4.343 | 2.31 × 10^-5^ |
| CTX:Medium | -8.117 ±3.540 | -2.293 | 0.023 |
| Resistance:Time | 5.03 × 10^-3^ ±1.11 × 10^-4^ | 4.540 | 1.01 × 10^-5^ |
| Resistance:Medium:Time | -4.37 × 10^-3^ ±6.54 × 10^-4^ | -6.681 | 2.70 × 10^-10^ |

^a^ Null deviance (191 d.f.) = 72.138, residual deviance (185 d.f.) = 27.647. ^b^SEM = standard error of the mean
