# Supplementary material for: The Fitness of Beta-Lactamase Mutants Depends Nonlinearly on Resistance Level at Sublethal Antibiotic Concentrations
Source: mBio. 2023 Apr 27;14(3):e00098-23. doi: 10.1128/mbio.00098-23 (PMC10294655; doi:10.1128/mbio.00098-23)
Supplement: TABLE S2 [file mbio.00098-23-s0008.docx]

**Table S2: Primers used during amplicon sequencing.**

| Sample | Focal genotype of competition | Forward Primer | Reverse Primer |
| --- | --- | --- | --- |
| 1 | TEM*-1* | ACAAGGTCGCCGCATACACTATTCTC | ACAACCAGTGCTGCAATGATACCG |
| 2 | M182T | AGTAGGTCGCCGCATACACTATTCTC | AGTACCAGTGCTGCAATGATACCG |
| 3 | E104K | TGATGGTCGCCGCATACACTATTCTC | TGATCCAGTGCTGCAATGATACCG |
| 4 | G238S | TTGAGGTCGCCGCATACACTATTCTC | TTGACCAGTGCTGCAATGATACCG |
| 5 | E104K M182T | ATCTGGTCGCCGCATACACTATTCTC | ATCTCCAGTGCTGCAATGATACCG |
| 6 | M182T G238S | GCTAGGTCGCCGCATACACTATTCTC | GCTACCAGTGCTGCAATGATACCG |
| 7 | E104K G238S | TCATGGTCGCCGCATACACTATTCTC | TCATCCAGTGCTGCAATGATACCG |
| 8 | E104K M182T G238S | TAATGGTCGCCGCATACACTATTCTC | TAATCCAGTGCTGCAATGATACCG |
| 9 | All above | TATAGGTCGCCGCATACACTATTCTC | TATACCAGTGCTGCAATGATACCG |

Note: The underlined sequence (the first four 5’ nucleotides) is the barcode used to allow demultiplexing and allocation of reads to the original sample. Amplicons made with the below primers were pooled prior to Truseq library preparation.
